# Ketamine Infusion Associated with Improved Neurology in a Patient with NMDA Receptor Encephalitis

**DOI:** 10.1155/2013/383125

**Published:** 2013-08-19

**Authors:** Michael MacMahon, Morag R. Naysmith, Stephanie McCallion, Jonathan Rhodes

**Affiliations:** ^1^Western General Hospital, Crewe Road South, Edinburgh EH4 2XU, UK; ^2^University of Edinburgh Medical School, 47 Little France Crescent, Edinburgh EH16 4TJ, UK

## Abstract

A young lady was ventilated on intensive care for a prolonged period with NMDA receptor encephalitis. She had undergone steroid, immunoglobulin, and plasmapheresis with no evidence of recovery. Her main management issue was the control of severe orofacial and limb dyskinesia. Large doses of sedating agents had been used to control the dystonia but were ineffective, unless she was fully anaesthetised. The introduction of a ketamine infusion was associated with a dramatic improvement in her symptoms such that it was possible to remove her tracheostomy two days after commencement. She was discharged shortly after that and is making a good recovery. The successful use of ketamine has not previously been described in this context, and we hope this case report will provide some insight into the management of this rare but serious condition.

## 1. Introduction

We would like to report a strong temporal relationship between the commencement of ketamine sedation and a dramatic improvement in the clinical features of NMDA receptor encephalitis.

## 2. Case **Presentation**

A previously fit and healthy 21-year-old female presented to the psychiatric services with uncharacteristic behaviour. She was noted to be extremely anxious, with repetitive phrases and evidence of disordered thought. She was admitted to a psychiatric hospital and treated for acute psychosis. However, her care was transferred to the acute medical services following the development of acute dystonia of the face, tongue, and symmetrical jerking movements of the limbs. This progressed over the next 48 hours to what appeared to be atypical generalised seizures while still maintaining some degree of volitional control, with a documented Glasgow Coma Scale between 3 and 11. Autonomic dysfunction was also evident with hyperpyrexia and varying tachycardia.

She was intubated and ventilated for airway protection. CT imaging was unremarkable, and an EEG, although was encephalopathic, did not show status epilepticus. CSF was not pleocytic with normal biochemistry. Initially, she was treated for atypical status epilepticus with propofol, phenytoin, and midazolam; however, ongoing seizure-like activity and orofacial dyskinesia were noted with no EEG correlate.

A provisional diagnosis of NMDA receptor encephalitis was made by the neurology team, and although a serum anti-NMDA receptor antibody assay was negative, treatment with methylprednisolone was commenced. The main clinical problem for the ICU team was managing her profound dyskinesia and agitation without acquiring iatrogenic complications such as ventilator-associated pneumonia, central line-associated infections, rhabdomyolysis, venous thrombosis, and propofol infusion syndrome while managing her dyskinesia and awaiting the response to immunotherapy.

Despite multiple adjuvant sedation regimes (in addition to propofol and alfentanil) including benzodiazepines, clonidine, dexmedetomidine, and risperidone, she remained either completely anaesthetised or unmanageable from distressing orofacial dyskinesia (tongue protrusion, drooling, and chewing), agitation, and coughing. Occasionally, she was responsive and able to follow one step commands but was never lucid. She developed early flexion contractures of her arms and legs, and peripheral access was not possible.

Over the course of her two month admission, she developed a probable ventilator-associated pneumonia and central line infection (both recognised early and treated successfully).

Although a serum anti-NMDA receptor antibody assay was negative, a subsequent CSF anti-NMDA receptor antibody test was positive, and immunotherapy in the form of sequential steroids, immunoglobulins, and plasmapheresis was instituted. It took over two months to be completed, and there were no initial signs of response: she remained as described previously. A tracheostomy was performed following a failed attempt at extubation (primarily due to laryngeal oedema).

Several days after the final plasmapheresis session, levetiracetam and a ketamine infusion (20 mg/hour) were commenced. The commencement of the ketamine coincided with a dramatic improvement in her clinical state: within a few hours there was no more orofacial dyskinesia, and she was lucid for the first time since admission.

She was successfully decannulated two days later and discharged to a level 1 environment later that week. She remained on 20 mg per hour of intravenous ketamine on discharge from ICU, and this was tapered off over the course of two weeks with no clear relapse in symptoms.

She has made an excellent recovery and has no lasting neurological deficit. Interestingly, she has no recollection of the events in intensive care, a finding commonly seen in NMDA receptor encephalitis.

## 3. Discussion

NMDA receptor encephalitis is a relatively new diagnostic entity, with the first reported case by Dalmau et al. in 2007 [1]. Since then, there have been several case reports and case series by Dalmau et al. [2, 3] and a group in the University college London (UCL) [4]. There is a great deal of similarity between the presentations in all of these case series: predominantly young patients, psychiatric prodrome, development of seizure-like motor features, and orofacial dyskinesia. There is a preponderance of ovarian teratoma, and indeed once resected, this subgroup appears to have a prognostic advantage. However with improved early recognition and immunotherapy, outcomes do appear to be improving with over 80% of confirmed cases making a good recovery at two years after diagnosis [3].

In this case, ketamine was used to control dyskinesia and allow a reduction in other sedative medications. There was a very clear temporal relationship between ketamine commencement and clinical improvement, although levetiracetam had also recently been started and she had undergone plasmapheresis in the previous week. Ketamine has not been described as being useful in this situation—in the UCL case series [4], they refer to using ketamine in two cases but without obvious clinical improvement. Initially, it may seem illogical that ketamine would work in this scenario. Dalmau demonstrated a reversible reduction in postsynaptic dendritic expression of NMDA receptors by exposing neurones to CSF from patients with anti-NMDA-receptor encephalitis—suggesting that the antibodies damage or reduce NMDA receptor expression [2]. Ketamine acts as an antagonist at the NMDA receptor and would be expected to worsen this condition. However, the pharmacology of ketamine may be more complex than direct antagonism of all NMDA receptors. An animal study demonstrated that low-dose ketamine persistently enhanced the potentiation of synaptic transmission of a subgroup of hippocampal NMDA receptors, in this case enhancing antidepressant activity [5]. It may therefore be that ketamine may have some partial agonist effects on some of the NMDA receptor subgroups: this could possibly account for the observed pharmacological effects in our patient. We hope this report will stimulate interest in the potential use of ketamine in the supportive management of NMDA receptor encephalitis.
